# Rhamnolipid production by a gamma ray-induced *Pseudomonas aeruginosa* mutant under solid state fermentation

**DOI:** 10.1186/s13568-018-0732-y

**Published:** 2019-01-08

**Authors:** Ghadir S. El-Housseiny, Khaled M. Aboshanab, Mohammad M. Aboulwafa, Nadia A. Hassouna

**Affiliations:** Department of Microbiology and Immunology, Faculty of Pharmacy, Ain Shams University, Organization of African Unity St., Abbassia, POB: 11566, Cairo, Egypt

**Keywords:** *Pseudomonas aeruginosa*, Response surface methodology, Glycerol, Rhamnolipids, Solid state fermentation

## Abstract

Solid-state fermentation has a special advantage of preventing the foaming problem that obstructs submerged fermentation processes for rhamnolipid production. In the present work, a 50:50 mixture of sugarcane bagasse and sunflower seed meal was selected as the optimum substrate for rhamnolipid production using a *Pseudomonas aeruginosa* mutant 15GR and an impregnating solution including 5% v/v glycerol. Using Box–Behnken design, the optimum fermentation conditions were found to be an inoculum size 1% v/v, temperature 30 °C and unlike other studies, pH 8. These optimized conditions yielded a 67% enhancement of rhamnolipid levels reaching 46.85 g rhamnolipids per liter of impregnating solution, after 10 days, which was about 5.5 folds higher than that obtained by submerged liquid fermentation. Although maximum rhamnolipids concentration was obtained after 10 days of incubation, rhamnolipids concentration already reached high levels (41.87 g/l) after only 6 days. This rhamnolipid level was obtained in a shorter time and using lower carbon source concentrations than most studies reported so far. The findings obtained indicate an enormous potential for employing solid-state fermentation for rhamnolipid production by the studied isolate.

## Introduction

Rhamnolipids (RLs) are promising biosurfactants mainly used for environmental applications because of their impressive emulsifying and surface active properties. However, their use is limited because of their elevated costs relative to that of chemical surfactants (Noh et al. 2014). Research on RLs production was mainly directed to submerged liquid fermentation (SLF) until recently. This production method creates serious foaming problems which are expensive to combat (Sodagari and Ju 2014; Winterburn and Martin 2012). Instead, solid-state fermentation (SSF) which has great potential for RL production has been introduced (Singhania et al. 2009; Narendrakumar et al. 2017).

SSF is a biological process performed in the absence of free water; using a substrate having sufficient moisture to aid in microbial growth and metabolic activity. The solid substrate could either be an inert material supporting the microorganism’s growth on it or the source of nutrients (Thomas et al. 2013). The potential of SSF is to offer the microbes an environment very similar to the natural environment where they normally live. This is probably the main reason why higher product concentrations are obtained using SSF in comparison to SLF (Thomas et al. 2013). The substrates utilized in SSF are usually agro-industrial residues or by-products and this not only offers economic value to these wastes, but also resolves their disposal problem and therefore reduces pollution. Moreover, the use of these low cost residues makes the bioprocess economically attractive. Therefore, these environmental benefits have shifted the industrial manufacturing towards SSF due to the increased demand for ecofriendly processes rather than chemical processes (Thomas et al. 2013). Other advantages of the SSF over the SLF are: smaller volume of fermentor; removed stirring costs; lower sterilization energy costs; reduced product recovery costs; lower contamination risk since the environment is less favourable for many bacteria (Mussatto et al. 2012). Only a few studies on the RL production using SSF have been reported so far (Camilios-Neto et al. 2008, 2011). Accordingly, the present work aims at studying the various physiological parameters influencing RL production by *P. aeruginosa* mutant 15GR under SSF using Response Surface Methodology (RSM).

## Materials and methods

### Microorganism

The *P. aeruginosa* 15GR from Culture Collection Ain Shams University (CCASU) (strain number, CCASU-P15GR) is a RL hyperproducer mutant obtained by gamma radiation of *P. aeruginosa* isolate P6 (CCASU-P6) in our previous study (El-Housseiny et al. 2017). This isolate was preserved in Luria–Bertani (LB) broth (Lab M, Topley house, England) containing 20% glycerol at − 80 °C.

### Culture media

The mineral salts medium (MSM) (Bodour et al. 2003) containing 2% v/v glycerol as the sole carbon source (named GMSM) was prepared and used in this study. The pH of this media was adjusted to 7 using KOH pellets.

### Production of RLs

#### Seed culture preparation

A loopful from *P. aeruginosa* 15GR was inoculated into 25 ml trypticase soy broth contained in an Erlenmeyer flask (250 ml) and incubated overnight at 30 °C and 250 rpm. The resulting culture was centrifuged (10,000 rpm for 10 min) and the cells were then washed once and resuspended in GMSM to obtain a count of 5 × 10^9^ cfu/ml.

#### Production of RL by SSF using different solid substrates

Each Erlenmeyer flask (250 ml) contained 10 g of one or a mixture of two of the following solid substrates (dried at room temperature): sugarcane bagasse (residue remaining after extraction of sugarcane juice from sugarcane stalks obtained from a local market, chopped into small fragments), sunflower seed meal (sunflower seeds were obtained from a local market, grinded and passed through a mesh sieve with 1.4 mm openings), corn bran, soybean meal, wheat bran, rice straw (all obtained from a local market). In each case, the total initial dry mass was 10 g (Table 1). The flasks were then sterilized by autoclaving for 15 min at 121 °C. Impregnating solution used was GMSM and its used amount was different from one substrate to another, depending on the substrate’s liquid absorption capacity (see Table 1) (Camilios-Neto et al. 2011). This solution was inoculated with 0.4 ml of seed culture, and mixed with the solid substrate (final bacterial concentration = 2 × 10^9^ cfu per 10 g solid substrate). The flasks were then incubated at 30 °C for 6 days without agitation. Control flasks containing the different substrates were treated similar to the test ones but were left uninoculated (Camilios-Neto et al. 2011).

**Table 1:** Different solid substrates used and their liquid absorption capacity (ml) of impregnating solution

Solid substrate (mixtures contain 50% of each substrate by mass)	Liquid absorption capacity (ml) of impregnating solution per 10 g of solid substrate
Sugarcane bagasse	25
Corn bran	17
Sunflower seed meal	15
Soybean meal	15
Wheat bran	15
Rice straw	20
Sugarcane bagasse + corn bran	20
Sugarcane bagasse + sunflower seed meal	20
Sugarcane bagasse + soybean meal	20
Sugarcane bagasse + wheat bran	20
Sugarcane bagasse + rice straw	20
Sunflower seed meal + corn bran	20

### Extraction of RLs

Aliquots of 50 ml of distilled water were added to each flask at the end of the incubation period and these flasks were agitated for 1 h at 30 °C and 200 rpm. The obtained suspension was filtered through gauze pieces, and the remaining liquid was manually squeezed out then added to the filtrate. The whole process was repeated twice (Camilios-Neto et al. 2011). The filtrates were then pooled and centrifuged for 10 min at 10,000 rpm to collect the supernatant. In case of sunflower seed meal and mixtures containing this substrate, supernatant was found to contain residual oil. Therefore, these supernatants were vigorously shaken with n-hexane 1:1 (v/v) to remove residual oil and centrifuged (10,000 rpm, 10 min) to separate the aqueous and n-hexane phases. This was done to avoid interference during orcinol assay (Kosaric and Vardar-Sukan 2014). The aqueous phase was then used for RL quantification. RL concentrations were expressed first as the product mass per kilogram of initial dry solids (g/kg IDS). In addition, to compare with results obtained in SLF, we expressed RL concentration as grams per liter of impregnating solution added to the solid substrate (g/l IS) (Camilios-Neto et al. 2011).

### Quantification of RLs

RL concentration was obtained using the modified colorimetric orcinol assay (Chandrasekaran and BeMiller 1980; Koch et al. 1991; Abdel-Mawgoud et al. 2009). First, RLs in the supernatant were extracted as explained by Wu and Ju (Wu and Ju 1998) using ethyl acetate. The separated organic phase was then evaporated at 80 °C and the resulting residue was dissolved in distilled H_2_O adjusted to pH 7 using 2.5 N NaHCO_3_. An aliquot of 900 μl orcinol reagent (0.19% orcinol in 53% H_2_SO_4_) was added to 100 μl of this aqueous extract and heated in a water bath (80 °C for 30 min). The mixture was allowed to cool to room temperature and the absorbance of the developed color (A_421_) was measured against blank (Daoshan et al. 2004). The concentration of RL in the supernatant was calculated from an equation of a calibration curve prepared using a standard RL (AgSciTech Inc., Logan, Utah, USA) (A_421_ nm = 0.0047 × RL concentration), considering the dilution factor (D.F.) of the diluted aqueous extract, as follows:

$${\text{Concentration of RL }}\left( {{\text{mg}}/{\text{l}}} \right)\, = \,\left( {{\text{A}}_{ 4 2 1} /0.00 4 7} \right)\, \times \,{\text{D}}.{\text{F}}.$$

### Studying the different factors affecting RL production by *P. aeruginosa* 15GR using SSF

#### Studying the time course of RL production in SSF using the selected substrate (sugarcane bagasse and sunflower seed meal) and comparing it to the production in SLF

Six Erlenmeyer flasks containing the selected substrate were prepared. Twenty milliliters impregnating solution (GMSM) inoculated with 0.4 ml of seed culture (2% v/v) was mixed with the solid substrate and these flasks were then incubated at 30 °C. Over an incubation period of 12 days, one flask was removed at specific time intervals for extraction and determination of RL concentration. One flask was left uninoculated and served as a control. To compare between SSF and SLF, the production process was also carried out in 250 ml Erlenmeyer flasks containing 50 ml of GMSM. These flasks were inoculated with the seed culture prepared above (2% v/v) and incubated at 250 rpm and 30 °C. At specified time intervals, samples were taken from the fermentation broth for RL quantification.

### Effect of agitation rate

In these experiments, two flasks were prepared as described above for SSF; one was incubated at 30 °C without agitation and the other incubated at 30 °C with an agitation rate of 250 rpm. After incubation, RLs were extracted as described. Control uninoculated flasks were prepared and treated similarly.

### Effect of using variable concentrations of glycerol in impregnating solution

Flasks (250 ml) containing sugarcane bagasse and sunflower seed meal were prepared and sterilized. Twenty milliliters aliquots of MSM containing different concentrations of glycerol (2%, 5%, 10% v/v) were inoculated with seed culture (2% v/v), mixed with the solid substrate and incubated at 30 °C. After incubation, RLs were extracted as described above. Control uninoculated flasks were prepared and treated similarly.

### Response surface methodology (RSM) for optimizing RL production using SSF

Factors such as inoculum size (represented by the code A), temperature (represented by the code B) and pH (represented by the code C) were optimized by RSM. Experimental Box–Behnken design (BBD) was employed and the factors and levels used for these experiments were: inoculum size of 1, 2 or 5% v/v; temperature of 30, 33.5 or 37 °C; and pH of 6, 7 or 8. A total of 13 runs were carried out with 1 centerpoint, each having an uninoculated control treated similarly. One response value, the RL concentration (RL, g/l) was measured accordingly after 10 days of incubation. The design of experiments was carried out by Design Expert^®^ v. 7.0 (DesignExpert ^®^ Software, Stat-Ease Inc., Statistics Made Easy, Minneapolis, MN, USA).

### Experimental verification of RSM results

A new SSF experiment was performed using optimal culture conditions recommended by the numerical optimization function in the Design Expert software. The RL production was measured and compared with results predicted by the model.

### Studying the time course of RL production by *P. aeruginosa* 15GR under optimized conditions

Six Erlenmeyer flasks containing 10 g of sugarcane bagasse and sunflower seed meal mixture were prepared. Twenty milliliters of impregnating solution (MSM + glycerol 5%) inoculated with 1% v/v of seed culture was mixed into the solid substrate and these flasks were incubated at 30 °C, initial pH 8 with no agitation. Over an incubation period of 12 days, one flask was removed at specific time intervals for extraction and determination of RL concentration. One flask was left uninoculated and served as a control.

## Results

### Production of RLs by SSF using different solid substrates

**Fig. 1:**
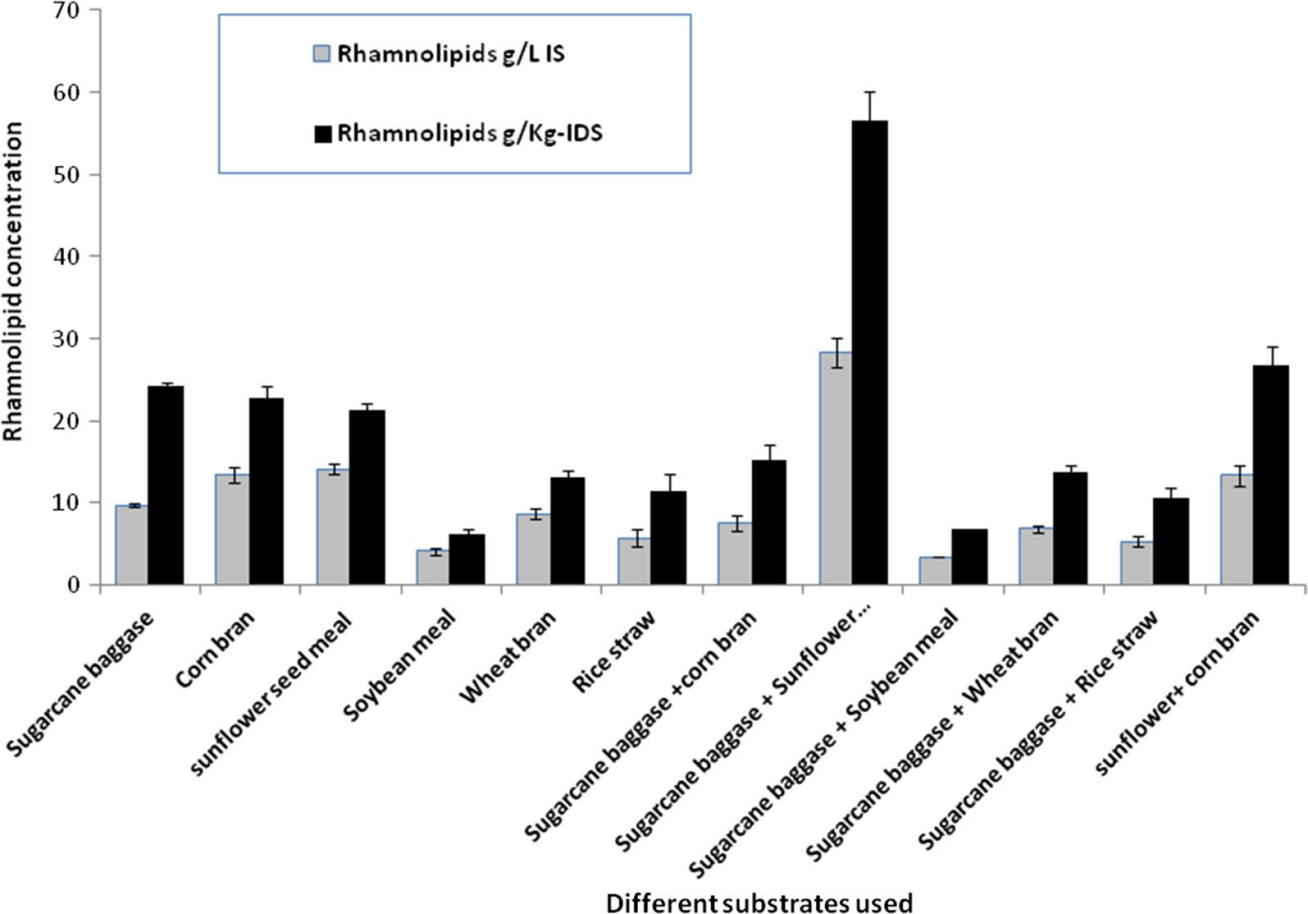
Effect of different solid substrates on RL production by *P. aeruginosa* 15GR in SSF after 6 days of incubation at 30 °C. Values plotted are the means of triplicate results while error bars indicate the standard deviation of the data

As shown in Fig. 1, sugarcane bagasse, sunflower seed meal and corn bran gave the highest results as single substrates. Note that these three flasks produced large amounts of foam during extraction. However, when comparing the RL production in the different mixtures tested, the highest RL level of 28.25 g/l IS (56.5 g/kg IDS) was obtained with the mixture of sugarcane bagasse and sunflower seed meal after 6 days of incubation and this flask produced the highest foam up on extraction. Therefore, this solid state mixture was chosen for further experiments.

### Different factors affecting RL production by *P. aeruginosa* 15GR using SSF

#### Time course of RL production in SSF using the selected substrate (sugarcane bagasse + sunflower meal) and in SLF

**Fig. 2:**
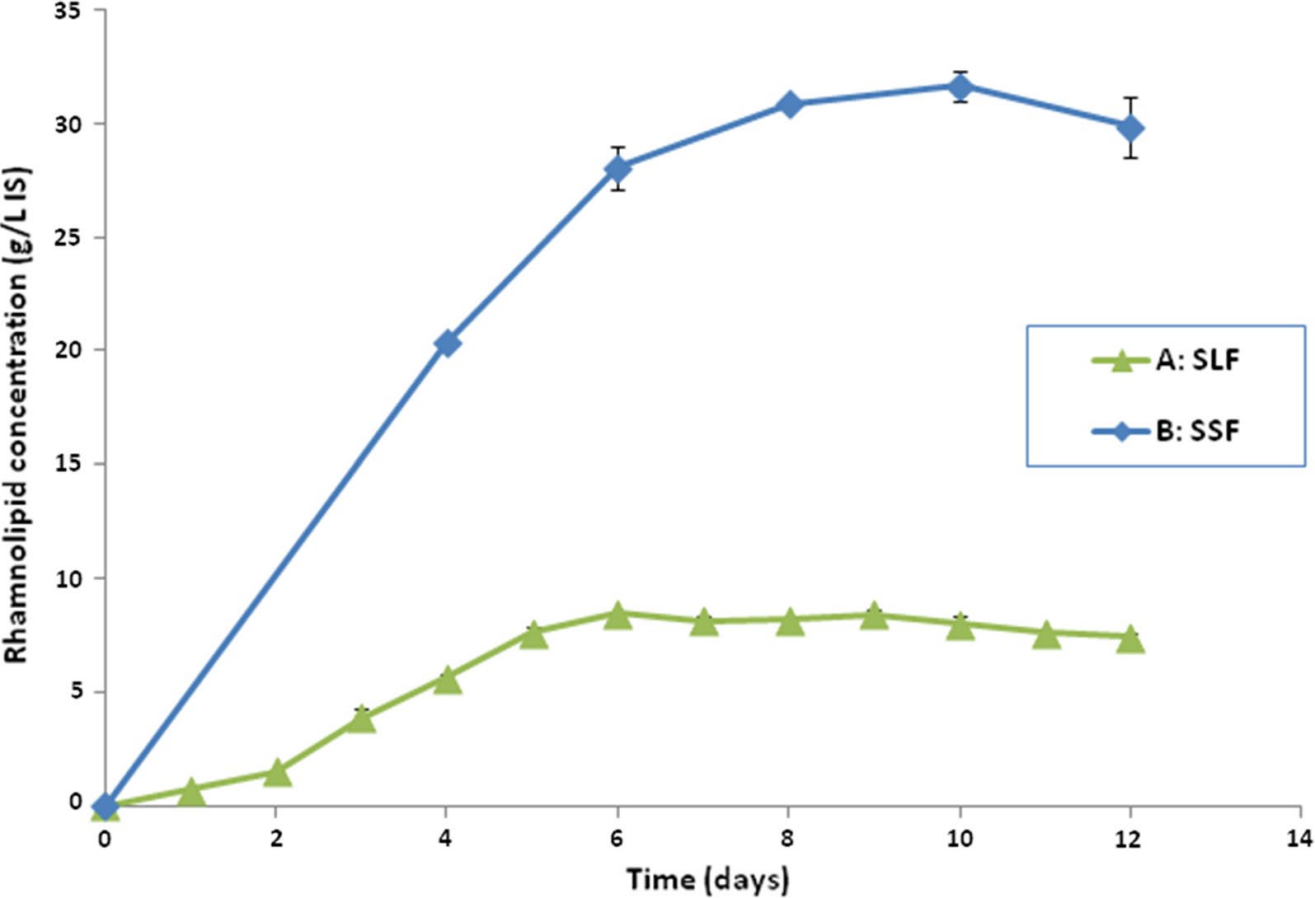
Time course of RL production by *P. aeruginosa* 15GR in **a** SLF using GMSM culture media, 30 °C and 250 rpm; **b** SSF of a 50:50 mixture of sugarcane bagasse and sunflower seeds, using GMSM as an impregnating solution. Values plotted are the means of triplicate results while error bars indicate the standard deviation of the data

Figure 2 showed the profile of RL production in both SLF and SSF. In case of SSF, the RL level increased linearly at the beginning, to reach 28 g/l-IS (56 g/kg-IDS) after 6 days of incubation. This level continued to increase, but slowly, reaching 31.65 g/l-IS (63.3 g/kg-IDS) after 10 days of incubation and a decline in RL production was observed after further incubation. Therefore, results in subsequent experiments were obtained after 10 days of incubation.

Using SLF, maximum RL production by *P. aeruginosa* 15GR was obtained at day 6, reaching 8.45 g/l only (Fig. 2).

### Effect of agitation rate

RL production in SSF approach using an agitation rate of 250 rpm resulted in a RL production of 30.5 ± 0.25 g/l-IS. Therefore, no shaking was used in subsequent experiments since no significant change was obtained as compared to that with no shaking (31.5 ± 0.5 g/l-IS).

### Effect of variable concentrations of glycerol in impregnating solution

**Fig. 3:**
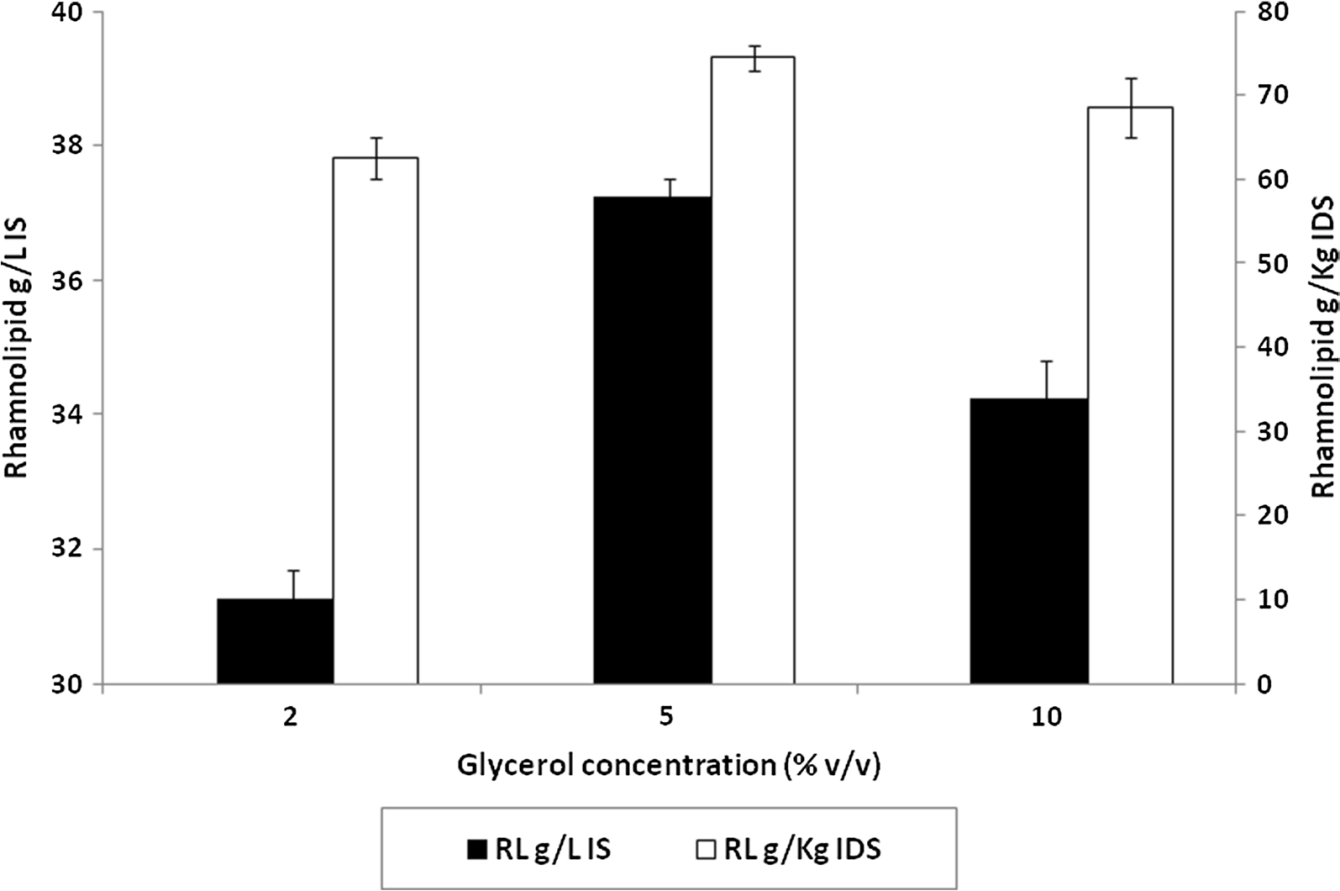
Effect of variable glycerol concentrations on RL production by *P. aeruginosa* 15GR in SSF. Values plotted are the means of triplicate results while error bars indicate the standard deviation of the data

Flasks containing different concentrations of glycerol contained in impregnating solution were tested. As delineated in Fig. 3, 5% v/v glycerol resulted in the highest RL production of 37.25 g/l-IS (74.5 g/Kg IDS) after 10 days incubation at 30 °C. Therefore, this concentration was used for further experiments.

### Response surface methodology (RSM) for optimizing RL production using SSF

After carrying out the experiments suggested by Design Expert software, the observed responses were recorded (Table 2). From these responses, the software automatically suggests a model which is a good-fitting mathematical function relating the response with the input factors tested. Predicted responses are then calculated from this fitted equation (Table 2) and used to estimate residuals and construct statistical and graphical summaries by the Design Expert software. The coefficients in this equation compensate for the differences in the ranges of the factors as well as the differences in the effects. These coefficients cannot be intuitively interpreted due to their dependence on the scaling of the factor levels (https://www.statease.com/pubs/handbk_for_exp_sv.pdf). This fitted equation is given by Eq 1:

1 $${\text{RL}}\, = \, 80.97796-1.74507*{\text{A}}-2.3*{\text{B}}\, + \, 4.50625*{\text{C}}$$

**Table 2:** Experimental Box–Behnken design (BBD) with the actual values of the independent factors inoculum size (A), temperature (B) and pH (C) and the observed and predicted responses

Run no.	Inoculum size (%)	Temperature (°C)	pH	Response RL concentration (g/l)	
				Observed	Predicted
1	1	30	7	39.15 ± 0.84	41.78
2	2	30	8	43.85 ± 0.28	44.54
3	2	30	6	35.75 ± 1.63	35.53
4	5	30	7	34.15 ± 0.83	34.80
5	1	33.5	8	38.70 ± 1.35	38.23
6	1	33.5	6	32.40 ± 2.05	29.22
7	2	33.5	7	36.35 ± 0.98	31.98
8	5	33.5	8	29.95 ± 0.03	31.25
9	5	33.5	6	23.00 ± 0.30	22.24
10	1	37	7	23.50 ± 1.00	25.68
11	2	37	8	30.10 ± 0.55	28.44
12	2	37	6	15.40 ± 0.20	19.43
13	5	37	7	19.50 ± 0.50	18.70

ANOVA results are displayed in Table 3. The Model F-value of 36.99 for RL production implied the significance of the model, since there is only a 0.01% chance that this large “Model F-Value” could result due to noise (P value < 0.0001). Moreover, A, B and C were significant factors (Table 3). Low coefficient of variation (CV) value of 8.62% was obtained which indicates that the experimental values were of adequate reliability. The coefficient of determination R^2^ was 0.9250, indicating that 92.50% of variability in the response can be interpreted by the model. The Predicted R-Squared (Pred R^2^) of 0.8620 was in acceptable agreement with the Adjusted R-Squared (AdjR^2^) of 0.9. An adequate precision ratio of 17.487 was recorded which suggested an adequate signal and that the present model could be used to navigate the design space.

**Table 3:** The analysis of variance (ANOVA) for the response surface linear model regarding RL concentration (RL)

Source	Sum of square	Degree of freedom	Mean square	F-value	P value Prob > F
Model	787.69	3	262.56	36.99	< 0.0001
A	106.82	1	106.82	15.05	0.0037
B	518.42	1	518.42	73.04	< 0.0001
C	162.45	1	162.45	22.89	0.0010
Residual	63.88	9	7.10		
Corrected total	851.56	12			

**Fig. 4:**
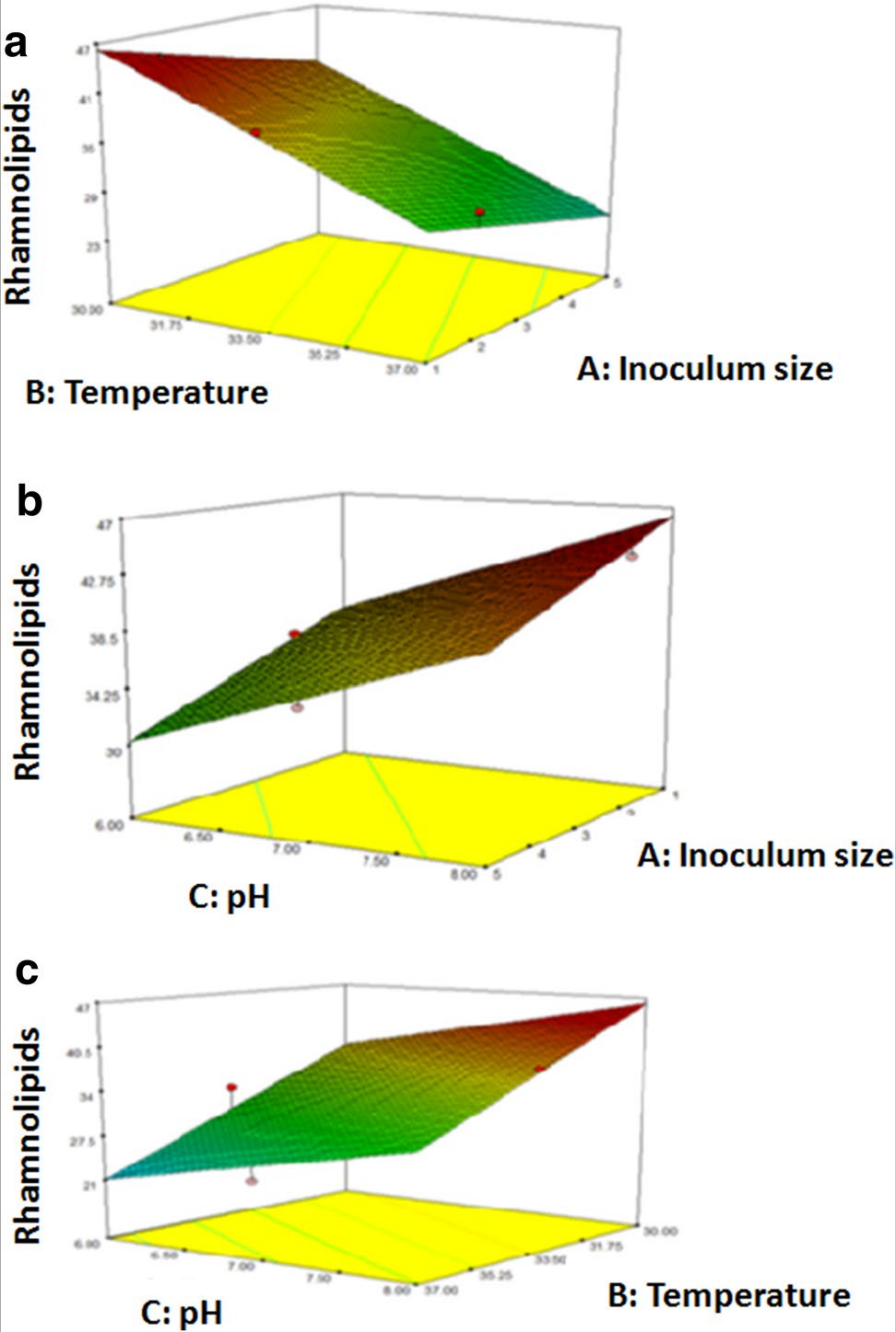
Three dimensional (3D) surface plots for the effects of temperature, inoculum size and pH on RL production by *P. aeruginosa* 15GR using SSF (obtained from Design Expert software). **a** pH is fixed at 8, **b** Temperature is fixed at 30 °C, **c** Inoculum size is fixed at 1% v/v

The 3D plots between the input factors are shown in Fig. 4. From these plots and using numerical optimization function in the Design expert software, optimum conditions for maximum RL production were found to be an inoculum size of 1%, temperature of 30 °C and pH of 8.

### Experimental verification test

Using these recommended optimum levels of the three factors (30 °C, 1% and pH 8), RL concentration reached 46.85 g/l-IS. This value was very similar to the value predicted by the model (46.28 g/l-IS) which reflects the accuracy and usefulness of the RSM to optimize the RL production process.

### Model diagnostics

**Fig. 5:**
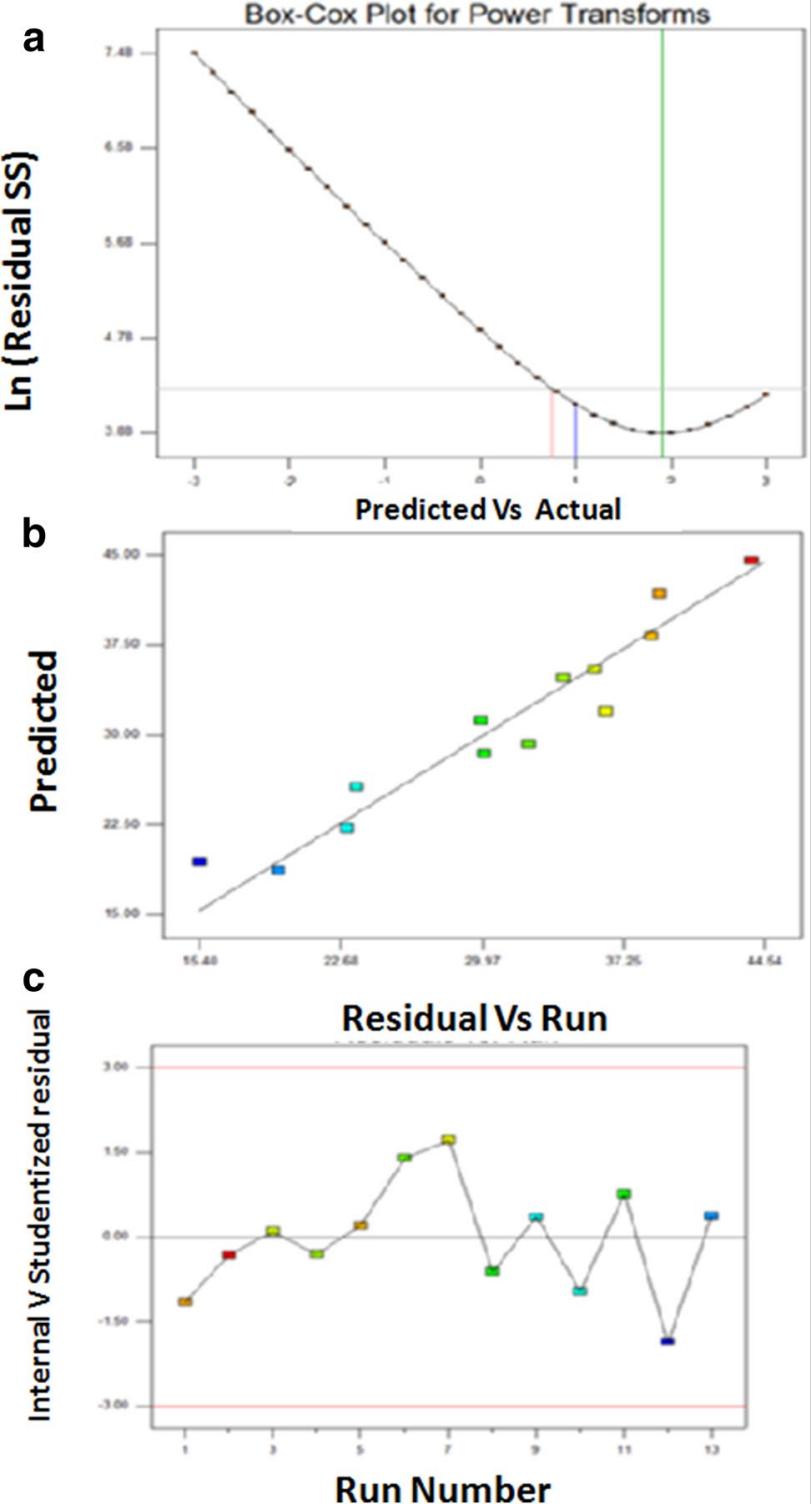
Diagnostic plots for the effects of temperature, inoculum size and pH on RL production by *P. aeruginosa* 15GR using SSF (obtained from Design Expert software) **a** Box–Cox plot. **b** Predicted vs. actual plot. **c** Residuals vs. run plot
- *The predicted versus actual plot* As shown in Fig. 5b, the values were distributed close to the straight line, which implied that actual and predicted values were very close to each other.
- *Residuals vs Run plot* showed that the points are scattered around zero suggesting that the model fit the data (Fig. 5c).

- *Box Cox plot* The Box–Cox plot showed that no further transformation was needed and the model was proven to be sufficient (Fig. 5a).

### Time course of RL production by *P. aeruginosa* 15GR under optimized conditions

**Fig. 6:**
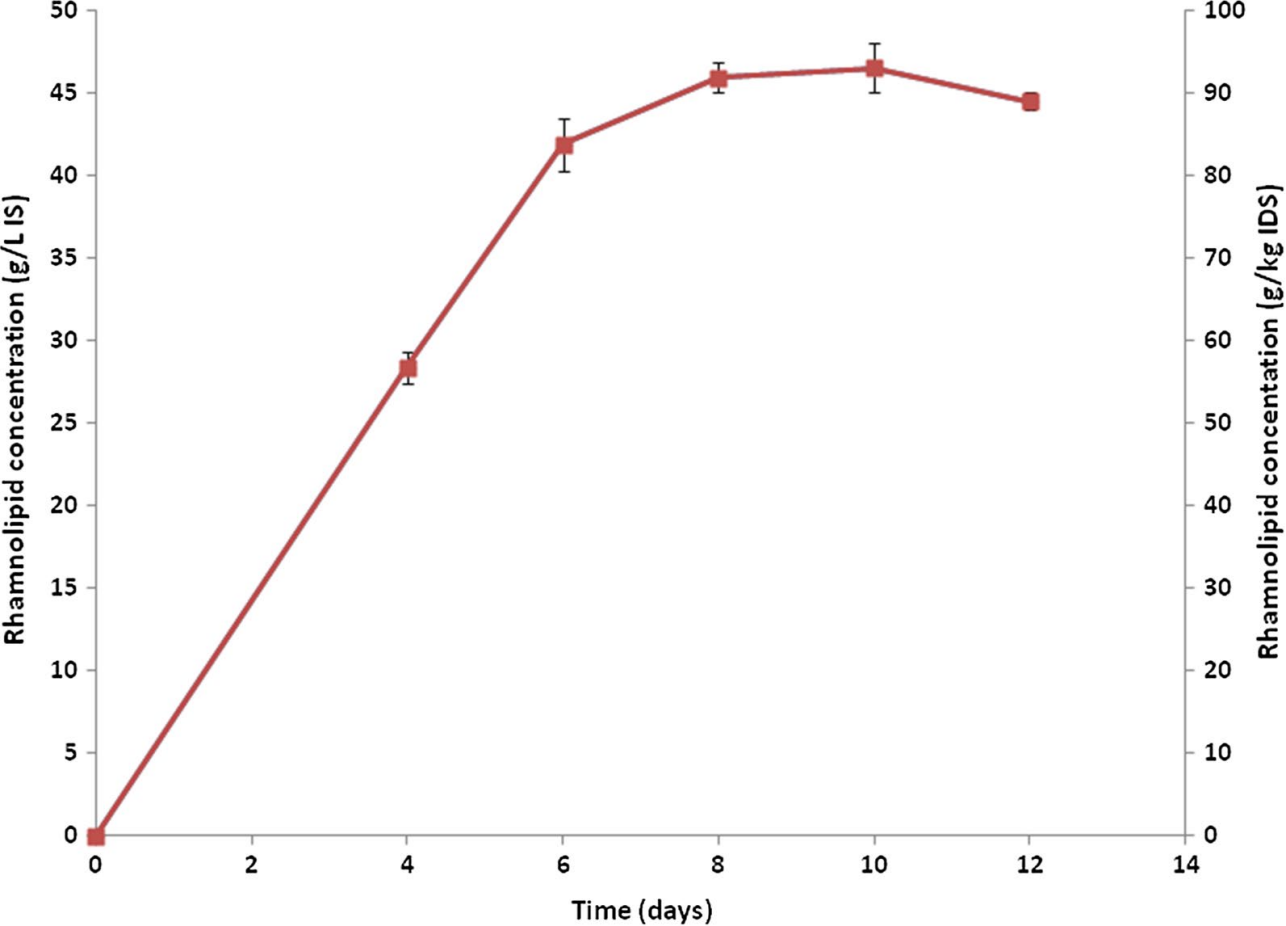
Time course of the RL production by *P. aeruginosa* 15GR in SSF using optimized conditions. Values plotted are the means of triplicate results while error bars indicate the standard deviation of the data

The time course profile, using a 50:50 mixture of sugarcane bagasse and sunflower seed meal, supplemented with impregnating solution containing 5% v/v glycerol using optimized conditions (temperature 30 °C, inoculum size 1% v/v and pH 8) was tested. As shown in Fig. 6, production increased rapidly during the first 6 days of incubation, then a slight increase was noticed at day 10. However, the RL concentration this time was higher (46.85 g/l-IS (93.7 g/kg-IDS)) than the original process (31.65 g/l-IS (63.3 g/kg-IDS)).

## Discussion

Recently, SSF has built up reliability in many industries and has evolved as an interesting substitute to SLF (Singhania et al. 2009). Severe foaming problems usually result from the production of biosurfactants in SLF and therefore, researches have suggested their production in SSF. Of these biosurfactants, RLs have been the most attractive for their production in SSF in recent years. The present work aimed at the optimization of the fermentation conditions for RL production in SSF. Twelve different solid substrates or combinations of solid substrates were screened for the production of RLs by the hyperproducing mutant 15GR using GMSM as the impregnating solution. Selection of an appropriate substrate is an important feature of SSF since it acts as both a source of nutrients and a physical support (Pandey 2003). As shown in the results, the highest RL level was obtained with the mixture of sugarcane bagasse and sunflower seed meal. Sugarcane bagasse, a porous residue obtained from cane stalks after the juice extraction from sugarcane (Soccol et al. 2010) consists chiefly of cellulose and hemicelluloses, lignin, nitrogenous compounds, and ash (Abdullah et al. 2006). Sunflower seed meal is obtained by grinding sunflower seeds which are rich in lipids, carbohydrates and proteins (Alberton et al. 2010). The high RL levels obtained using this mixture may be due to the fact that oils usually stimulate RL production, as reported in previous SLF studies (Benincasa and Accorsini 2008; Costa et al. 2006; Trummler et al. 2003). Another explanation may be that the mixture of substrates resulted in a substrate bed with properties superior to single substrates. Using sunflower seed meal alone caused the solid substrate to compact considerably probably due to its high lipid content. Sugarcane bagasse acts as a bulking agent, improving the substrate bed properties (Alberton et al. 2010).

After screening for the best substrate, our first experiment was a kinetic study to find out the time required for maximum RL production. As shown in the results, a maximum RL concentration of 31.65 g/l-IS (63.3 g/kg-IDS) was obtained after 10 days of incubation. Therefore, results in subsequent experiments were obtained at this time. Upon comparing RL production in SSF with that resulting from SLF using the same impregnating solution as culture media, it was found that SSF (using sugarcane bagasse and sunflower seed meal) resulted in over a threefold increase in RL production, which further proves the superiority of this process.

In an attempt to improve RL production, the effect of increasing the glycerol concentration in the impregnating solution was tested. As shown in the results, the highest RL yield (37.25 g/l-IS (74.5 g/Kg-IDS)) was observed with 5% v/v glycerol in the impregnating solution after 10 days incubation at 30 °C.

To optimize the culture conditions required for maximum RL production using SSF, RSM, the most efficient and straight forward statistical approach that permits concurrent measurement of several process variables, was carried out (Chen et al. 2012). Box–Behnken experimental design was chosen to optimize 3 factors; inoculum size, temperature and pH. The Box–Behnken design (BBD) is a convenient approach to find out the effects of different factors and their interactions on the responses. It usually takes three levels of each factor and all the design points lie within the safe operating region. The advantages of BBD are that it is considered to be more efficient, more powerful, requires fewer experimental runs than other designs such as Central Composite Design and three-level full factorial design, and hence is cheaper (Marasini et al. 2012).

ANOVA verifies the adequacy of the models and the P value is used as a tool to determine the significance of each of the studied factors. ANOVA results suggested that the model equation derived is significant and could adequately be used to describe the RL production by SSF (P value < 0.0001). Low coefficient of variation (CV) indicates that the experimental values were of adequate reliability. The CV reveals the precision level with which the treatments are compared, and the experiment reliability decreases as the CV value increases (Ghribi et al. 2012). Adequate (Adeq) Precision measures the signal to noise ratio, and a ratio more than 4 is commonly preferable (Abdel-Hafez et al. 2014). An adequate precision ratio of 17.487 in our study suggested an adequate signal and that the present model could be used to navigate the design space and could adequately be used to describe the RL production by SSF with *P. aeruginosa* 15GR.

The 3 D plots are plots that present details about the interaction between two factors and permit a simple prediction of the optimal conditions (Ghribi et al. 2012). From these plots and using numerical optimization function, optimum conditions for maximum RL production were found to be an inoculum size of 1%, temperature of 30 °C and pH of 8, resulting in a RL concentration of 46.85 g/l IS. The obtained model diagnostic plots also proved the validity of the model constructed in this study.

ANOVA results also revealed that all three factors had a significant effect on RL concentration. A higher inoculum size usually enhances microbial growth and other associated microbial activities of the microorganism until a certain value after which there could be a decrease in microbial activity as a result of nutrient limitations (Kashyap et al. 2002). In the present study, optimum inoculum size was found to be 1%v/v. This may be due to the severe competition among bacteria when inoculum size was increased, leading to change in metabolism towards a survival pattern. Alternatively, this may be because an increase in the initial inoculum size stimulated an earlier initiation of RL production instead of increasing cell concentration, which resulted in lower final biomass and hence lower final RL concentrations. Another critical factor affecting RL production is temperature. Wei et al. (2005) measured RL production and showed that 30 °C to 37 °C was the optimum temperature range. In this study, RL production reached a maximum at 30 °C. The pH also greatly influences many microbial metabolites production. Most of the previous studies reported that a pH range from 6 to 7 resulted in maximum RL production in different *Pseudomonas* species, depending on the strain used (Chen et al. 2007; Zhu et al. 2012). Moreover, Mulligan et al. (2014) reported that *P. aeruginosa* does not produce RLs at a pH higher than 7.5 and that a pH of 6.2 was optimum for RL production. Another study also showed that RL concentration decreased and reached its lowest point at a pH of 8 (Moussa et al. 2014). In contrast to these reports, in this study maximum RL production reached a maximum at an initial pH of 8. This result is in agreement with our previous study carried out on the parent isolate P6, where optimum pH for maximum RL production was also found to be 7.5 (slightly alkaline) (El-Housseiny et al. 2016). This suggests that optimum pH for maximum RL production is bacterial strain dependant and that the bacterial isolate used in this study was highly sensitive to pH for RL production. The commercial application of this powerful biosurfactant may thus be enhanced by reducing its production costs through increasing its yield using RSM.

Since major changes have been made in the fermentation conditions for RL production, the time course profile was repeated, using the optimized conditions reached (a 50:50 mixture of sugarcane bagasse and sunflower seed meal, an impregnating solution of 20 ml containing 5%(v/v) glycerol, inoculum size 1%v/v, pH 8 and incubation temperature of 30 °C). Again, maximum RL production was obtained after 10 days of incubation, however, the RL level this time was about 1.5 fold higher than results obtained in the previous time course study, reaching 46.85 g/l-IS. The obtained value was also about 5.5 folds higher than that obtained using SLF carried out in this study. Moreover, this RL level was obtained in a shorter time than most studies reported so far. In 2008, a maximum RL production of 46 g/l-IS (172 g/kg-IDS) by *P. aeruginosa* UFPEDA614 in SSF using a 50:50 mixture of sugarcane bagasse and sunflower seed meal with 37.5 ml of impregnating solution containing 10% v/v glycerol after 12 days was reported (Camilios-Neto et al. 2008). Although our RL yield is comparable with this value when expressed in terms of g/l-IS, our yield expressed in terms of g/kg of solid substrate showed lower values. This may be explained by the smaller volume of impregnating solution used in our study. Moreover, in 2011, the highest RL production of 45 g/l-IS was obtained by the same strain after 12 days of SSF using sugarcane bagasse and corn bran (1:1), and an impregnating solution of 35 ml containing 6% (v/v) of each of glycerol and soybean oil (Camilios-Neto et al. 2011). Therefore, in our study, the mutant 15GR yielded comparable RL concentrations that were reached in less time and using lower concentrations of carbon source than both these studies. Moreover, although maximum RL concentrations were obtained after 10 days of incubation, high RL levels were already achieved (41.87 g/l-IS (83.74 g/kg-IDS)) by the mutant 15GR after only 6 days of incubation, unlike Camilios-Neto et al. (2008), whose RL yields reached only about 24 g/l-IS (89 g/kg-IDS) after 6 days of incubation. In addition, in 2017, maximum RL yields of 18.7 g/l was obtained using glycerol as carbon source and rapeseed/wheat bran as matrix (Wu et al. 2017). Our study hence presents an appropriate basis for subsequent studies on RL production using SSF. In conclusion, these results showed that the application of BBD and RSM were successful in enhancing the RL production under SSF by 67% and a maximum RL concentration of 46.85 g/l-IS was obtained in the present study using a mixture of sugarcane bagasse and sunflower seed meal after only 10 days of incubation. Optimum fermentation conditions were found to be an inoculum size of 1%v/v, a temperature of 30 °C and a pH of 8. These results suggest that SSF may possibly be a feasible substitute for SLF to produce RLs since our maximum yield was comparable with values that have been obtained in SLF: 23.6 g/l (Noh et al. 2014), 32 g/l (Matsufuji et al. 1997), 36.7 g/l (Muller et al. 2011), and 46 g/l (Linhardt et al. 1989). These findings imply that RL production by *P. aeruginosa* 15GR in static tray bioreactors may be successful (Durand 2003). Additional concern to the application of SSF for RL production is hence justified for enhancing RL levels in laboratory-scale work even further and for moving up to pilot scale.
